# Species-Specific and Distance-Dependent Dispersive Behaviour of Forisomes in Different Legume Species

**DOI:** 10.3390/ijms22020492

**Published:** 2021-01-06

**Authors:** Maria K. Paulmann, Matthias R. Zimmermann, Linus Wegner, Aart J. E. van Bel, Grit Kunert, Alexandra C. U. Furch

**Affiliations:** 1 Plant Physiology, Matthias Schleiden Institute for Genetics, Bioinformatics and Molecular Botany, Faculty of Biological Science, Friedrich Schiller University Jena, Dornburger Straße 159, 07743 Jena, Germany; mpaulmann@ice.mpg.de (M.K.P.); Matthias.rudi.zimmermann@gmx.de (M.R.Z.); linus.wegner96@gmail.com (L.W.); 2 Department of Biochemistry, Max Planck Institute for Chemical Ecology, Hans-Knöll-Str. 8, 07745 Jena, Germany; gkunert@ice.mpg.de; 3 Interdisciplinary Research Centre, Institute of Phytopathology, Justus Liebig University, Heinrich-Buff-Ring 26, 35392 Giessen, Germany; Aart.v.Bel@bot1.bio.uni-giessen.de

**Keywords:** electrophysiology, forisome, *Medicago sativa*, *Pisum sativum*, sieve-tube occlusion, systemic signalling, *Trifolium pratense*, *Vicia faba*

## Abstract

Forisomes are giant fusiform protein complexes composed of sieve element occlusion (SEO) protein monomers, exclusively found in sieve elements (SEs) of legumes. Forisomes block the phloem mass flow by a Ca^2+^-induced conformational change (swelling and rounding). We studied the forisome reactivity in four different legume species—*Medicago sativa*, *Pisum sativum*, *Trifolium pratense* and *Vicia faba*. Depending on the species, we found direct relationships between SE diameter, forisome surface area and distance from the leaf tip, all indicative of a developmentally tuned regulation of SE diameter and forisome size. Heat-induced forisome dispersion occurred later with increasing distance from the stimulus site. *T. pratense* and *V. faba* dispersion occurred faster for forisomes with a smaller surface area. Near the stimulus site, electro potential waves (EPWs)—overlapping action (APs), and variation potentials (VPs)—were linked with high full-dispersion rates of forisomes. Distance-associated reduction of forisome reactivity was assigned to the disintegration of EPWs into APs, VPs and system potentials (SPs). Overall, APs and SPs alone were unable to induce forisome dispersion and only VPs above a critical threshold were capable of inducing forisome reactions.

## 1. Introduction

Long-distance transport in plants takes place in the vascular system, which is not only responsible for the nutrient distribution, but also for systemic dissemination of hormonal, electrical and macromolecular signals [1,2,3,4,5]. The conduits in vascular bundles of angiosperms are composed of serially arranged dead vessel elements as well as tracheids in the xylem and “semi-living” sieve elements (SEs) in the phloem [6,7]. Survival of the enucleate SEs is ensured by the support of adjacent companion cells (CC) [5,8] that are symplasmically connected to SEs by pore/plasmodesm units [9]. SEs are interconnected via plasma connections through perforations in the end walls (sieve pores) that allow unhindered mass flow [10].

Sieve pores can be closed in response to local damage or passing (electrical) alarm signals [11,12]. Two major sieve-pore sealing mechanisms prevent the loss of phloem sap from and invasion by pathogens into sieve tubes [13]. It seems that all angiosperm families constrict sieve pores by extracellular local deposition of callose [12,14,15]. A second mechanism is mediated through phloem (P-) protein plugs [16,17,18].

Structural P-proteins, some of which belong to the SEO (sieve-element occlusion) or the sieve-element occlusion-related (SEOR) family, come in a multitude of shapes [19,20,21,22,23]. Apart from the structural variants of loosely organized filaments [21,23], P-proteins also occur in densely packed, highly organized structures [24,25,26,27]. These spindle-shaped so-called forisomes [17,26] have only been found in the legume subfamily Papilionoideae [28]. Forisomes belong to the SEO-F family (sieve element occlusion by forisome) [29,30,31] and show a unique type of reactivity in response to Ca^2+^ elevation; they are strongly swelling (so-called dispersion) during a conformational transition from a fusiform to a rounded appearance [31,32]. Forisome dispersion produces instant occlusion of sieve plates as has been demonstrated in a few approaches [11,33,34]. Two types of forisomes have been described, forisomes with tail-like protrusions and tailless forisomes [24,28]. The main body of both types reacts to the same extent to surges in the Ca^2+^ concentration [28,35].

For an effective SE occlusion, the size of the forisomes compared to the SE diameter might be important [30]. Length and volume of the forisome body are related to the SEO-F-subunit composition which may vary with plant species [22,30]. For example, condensed forisomes in *Vicia faba* are wider and longer as compared to *Pisum sativum* and *Medicago truncatula* [30]. However, the question remained unanswered if the forisome dimensions are related to the reaction time-lapse after a stimulus (e.g., heat) in planta.

Forisome dispersion is mediated through passage of a stimulus-induced electrophysiological reaction (elR) and subsequent Ca^2+^ influx into the SE [11,13,33,36]. Different elRs have been described but their exact connection to forisome dispersion has been barely explored [33,37]. Heat-triggered electropotential waves (EPWs) that can induce forisome dispersion [33] are composed of different elRs which include action potentials (APs) and variation potentials (VPs) [37,38]. It was postulated that the waves of Ca^2+^ influx result from a concerted interaction between diverse Ca^2+^-permeable channels [11,13,37]. Which Ca^2+^-permeable channels are involved in the electrical propagation likely depends on the stimulus in question [13]. Probably, APs activate voltage-dependent or ligand-activated channels [39,40], VPs mechanosensitive or ligand-activated channels [13,41] and system potentials (SPs) have less influence on the Ca^2+^ level [42].

To get a broader knowledge about morphology and behavior of tailless forisomes four representatives of the subfamily Papilionoideae (*Medicago sativa*, *P. sativum*, *Trifolium pratense* and *V. faba*) were compared. It was investigated if the forisome size is intraspecifically and interspecifically related to the SE diameter, to which extent forisome reactivity is related to the forisome surface area, to which degree forisome dispersion depends on the distance from the heat stimulus, if dispersion and contraction are related in some way to the forisome size, which type of elR induced forisome dispersion, and if the readiness to dispersion is linked to the vascular orthostichy in *V. faba* plants.

## 2. Results

### 2.1. Forisome Surface Area (A_F_) Increases with SE Diameter

Being stopcocks, forisomes should be able to fully fill out the cross-sectional area of SEs [33,43] and their dimensions may vary as an adaptation to the SE dimensions. The dispersive state is the decisive factor for the efficiency of plugging. Unfortunately, the dimensions of the dispersed state cannot be measured, because dispersed forisomes are invisible with common light microscopy due to their optical properties [26]. Therefore, the dimensions of condensed forisomes are scored on the premise that the dimensions of the condensed state are approximately related to the volume of the dispersed state [44]. Furthermore, the diameter of SEs is taken as an easily amenable parameter, because this value is proportional to the cross-sectional area, which is to be blocked by the forisome.

**Figure 1:**
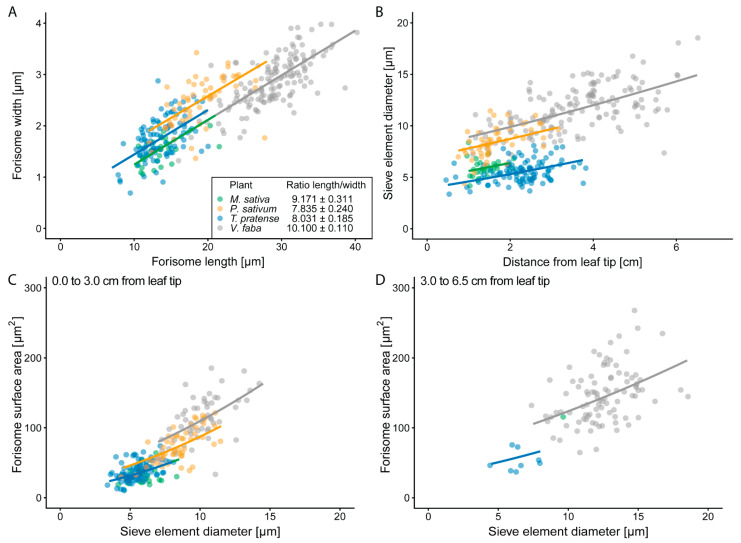
Relationships between sieve element (SE) diameter and forisome dimensions or distance from the leaf tip. (**A**) The relation between forisome width and forisome length and (**B**) between SE diameter and the distance from the leaf tip (**B**) is shown for the investigated legume species. The inset in (**A**) shows the ratio of forisome length to forisome width ± standard error as a numerical presentation of the graphs. (**C**,**D**) The forisome surface area (*A_F_*) was calculated based on the forisome width and length and related to the SE diameter. For a better graphical presentation, the relation between forisome surface area and SE diameter is shown for forisomes (**C**) close to (until 3 cm) and (**D**) further away from the leaf tip (3 cm to 6.5 cm). Statistical values are shown in Table S1. Replicates from different plants are presented in: Green—*M. sativa* (*n* = 27), orange—*P. sativum* (*n* = 53), blue—*T. pratense* (*n* = 97), and grey—*V. faba* (*n* = 147).

In general, forisome length and diameter are positively related (*p* < 0.001), but the precise relation varied with plant species (*p* < 0.001; Figure 1A; Table S1). *V. faba* and *M. sativa* forisomes were more slender than those of other species, whereas forisomes in *P. sativum* were the thickest (see inset in Figure 1A). The strength of the increase in forisome width with increasing length was the same for all plant species (*p* = 0.537; Figure 1A). SE diameters increased with the distance from the leaf tip (*p* < 0.001; Figure 1B; Table S1). At similar distances from the leaf tip, SE diameters differed between the species (*p* < 0.001; Figure 1B), *V. faba* had the largest and *T. pratense* the smallest SE diameters. The forisome surface area (*A_F_*) increased with the SE diameter and distance to the leaf tip (*p* < 0.001) in all species (Figure 1C,D; Table S1). At similar SE diameters and distances *A_F_* differed between the species (*p* < 0.001; Figure 1C,D) with the largest *A_F_* found in *V. faba* and the smallest in *T. pratense* and *M. sativa*. In conclusion, the parameters measured varied in quantity between species but not the relationship between the parameters.

### 2.2. The Closer to the Stimulus Site the Higher is the Chance of Full Forisome Dispersion

**Figure 2:**
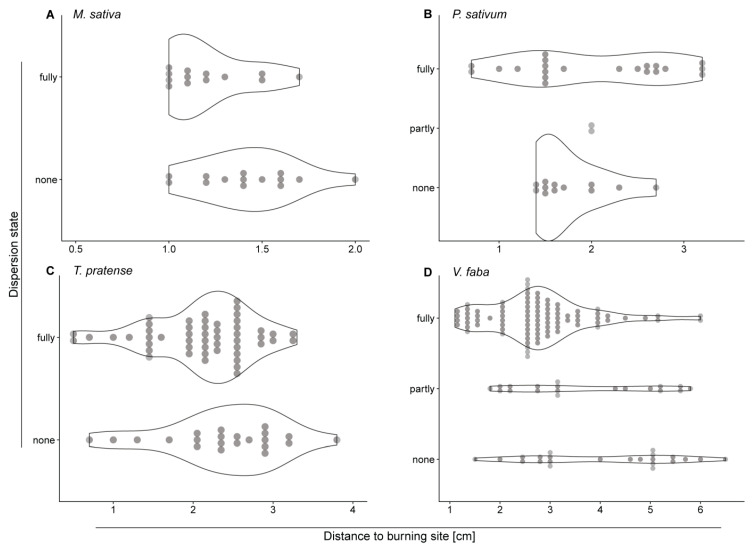
Effect of the distance from the stimulus on the forisome dispersion state (fully, partly, none) in experiments on the target leaflet. Depicted are violin plots with their width adjusted to the number of forisomes observed in a given state of dispersion at a given distance (each forisome indicated by a filled circle). The number of replicates varied between the species: *M. sativa* (**A**) *n* = 28, *P. sativum* (**B**) *n* = 35, *T. pratense* (**C**) *n* = 73, *V. faba* (**D**) *n* = 133. Statistical values are shown in Table S2.

To further elucidate the effect of the distance to the stimulus site on the rate of dispersion, the species were analyzed separately and different forisome dispersion states were distinguished (Figure 2). In the target leaflet of *V. faba,* the proportion of fully dispersed forisomes changed with the distance to the burning site (*p* < 0.001; Figure 2D). About 79% of the forisomes close to the burning site (until 3.0 cm) dispersed fully, whereas only 51% of the forisomes dispersed further than 3.0 cm away. For the other species the distance did not influence the dispersion state of the forisomes (*M. sativa*: *p* = 0.077; *P. sativum*: *p* = 0.472; *T. pratense*: *p* = 0.154; Figure 2; Table S2).

### 2.3. The Time Lapse until Full Forisome Dispersion Increases with Distance to Stimulus Site

**Figure 3:**
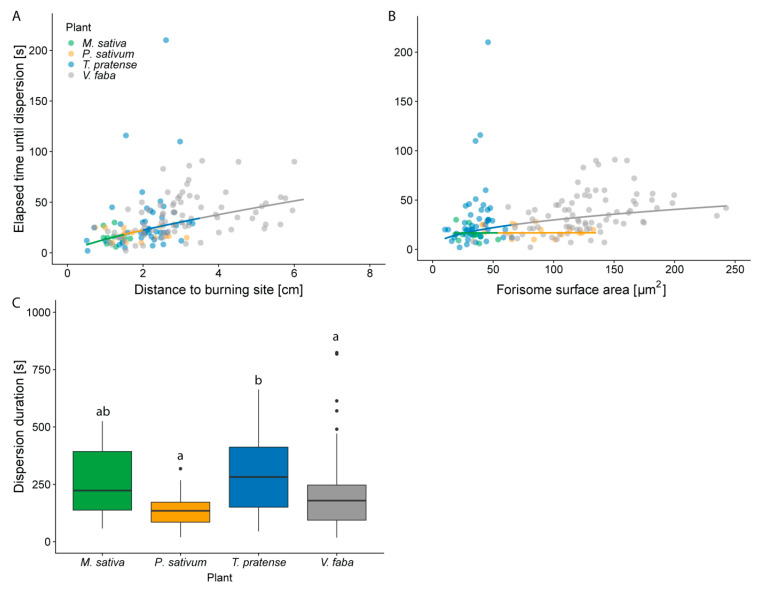
Influence of different parameters on the time lapse between heat stimulation and complete dispersion and on the dispersion time length. Only forisomes from experiments on the target leaflet were analysed. Both, (**A**) distance to the burning site and (**B**) the interaction of forisome surface area (*A_F_*) and species (**B**) have an effect on the time lapse between stimulus application and forisome dispersion. For easier visualization the results are shown in separate graphs. (**C**) Boxplot of the duration of dispersion associated with the species. Depicted are the median and the first and third percentile. Different letters indicate statistically significant differences. Statistical values are shown in Table S3. Replicates from different species (replicates for the time until dispersion; replicates for the duration of dispersion) are presented in different colours: green—*M. sativa* (*n* = 11; *n* = 11), orange—*P. sativum* (*n* = 16; *n* = 16), blue—*T. pratense* (*n* = 43; *n* = 36), and grey—*V. faba* (*n* = 77; *n* = 75).

The interspecific variation in *A_F_s* (Figure 1C,D) raised the question, if the reactivity (time lapse until maximal dispersion or length of the dispersion time) is affected by the forisome size. The time lapse from stimulation to full forisome dispersion increased with the distance from the burning site (from less than 10 s to over a minute), regardless of the species (*p* < 0.001; Figure 3A; Table S3). The effect of A_F_ on this time lapse depended on the species in question (*p* = 0.002; Figure 3B). Only in *V. faba* and *T. pratense,* the time lapse increased with increasing *A_F_* (Figure 3B; Table S3).

### 2.4. Plants Differ in the Duration of Forisome Dispersion

In contrast to the time lapse between stimulus and dispersion, the length of dispersion time was neither influenced by the distance from the stimulus (*p* < 0.261), nor by the *A_F_* (*p* = 0.341). The plant species, however, differed in forisome dispersion duration (*p* = 0.009; Figure 3C; Table S3). Forisomes of *T. pratense* remained the longest in the dispersed state and *V. faba* and *P. sativum* the shortest.

### 2.5. Distant and Systemic Stimuli Barely Induce Forisome Responses

**Figure 4:**
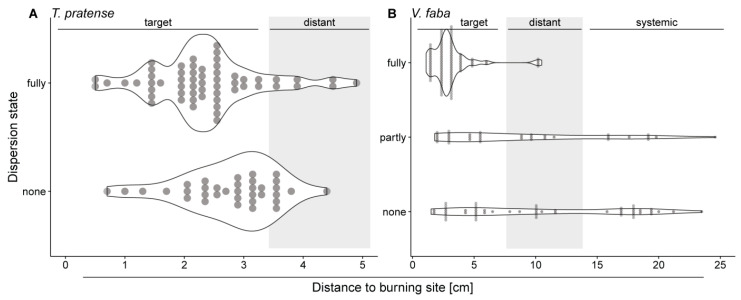
Effect of increasing distance and internode intermissions on forisome dispersion (fully, partly, none) after a heat stimulus to *T. pratense* (**A**) and *V. faba* (**B**). The width of the violin plots is adjusted to the count of forisomes observed in a given state at a given distance (each forisome indicated by a filled circle). For *T. pratense* 115, for *V. faba* 184 forisomes were monitored. Statistical values are shown in the text.

That the proportion of non-dispersive forisomes in target leaflets of *V. faba* increased with the stimulus distance (Figure 2D), incited to monitor heat-induced forisome reactions in distant (*V. faba* and *T. pratense*) and systemic leaflets (*V. faba*, compare Material and Methods). In both species, the proportion of forisomes that fully dispersed in response to heat stimuli decreased significantly in distant and systemic leaflets (*T. pratense*: *Χ²* = 6.712, *p* = 0.010; *V. faba*: *Χ²* = 54.584, *p* < 0.001; Figure 4). Whereas in the target leaflet the forisomes fully dispersed to 70% in *T. pratense* and 68% in *V. faba*, this proportion dropped to 35% in *T. pratense* (Figure 4A) and 17% in *V. faba* in distant leaflets (Figure 4B). After stimulation in *V. faba* none of the forisomes fully dispersed in systemic leaflets.

### 2.6. Forisome Dispersion is Only Triggered by a Variation Potential

**Figure 5:**
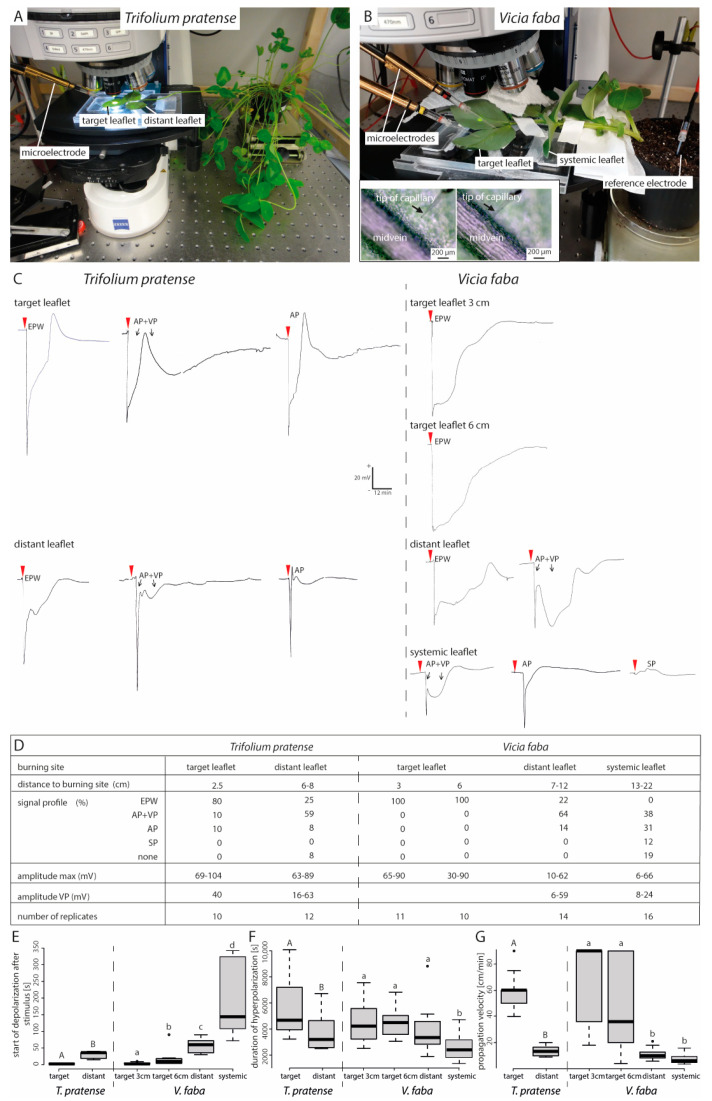
Electrophysiological reactions in response to local, distant and systemic heat stimulation of *V. faba* and *T. pratense* leaf tips. (**A**,**B**) for extracellular, electrophysiological measurements the tip of a glass capillary was pierced into lower side of the midvein of a *V. faba* or *T. pratense* target leaflet under microscopical surveillance to ensure a correct placement (see inset B). (**C**) Typical recordings of electrophysiological reactions at the target leaflet are shown for stimulation of target, distant and systemic leaflets. Scale bar in (**C**) is valid for all measurements. (**D**–**G**) Parameters of the recorded electrophysiological reactions in the respective leaflets are arranged side by side. (**E**) represents the start of the depolarization after the stimulus, (**F**) the duration of the depolarization and (**G**) the propagation velocity. Statistical values are shown in Tables S4 and S5. Different letters indicate significant differences. AP—action potential, VP—variation potential, SP—system potential, EPW—electropotential wave.

The decreased rate of forisome dispersion further away from the stimulus (Figure 3A and Figure 4) probably reflects a declining Ca^2+^ influx into the SEs along the elR propagation path. Ca^2+^-influx quantity is likely associated with the type of the passing elR [33]. To investigate the relationship between the mode of elR and forisome dispersion, elRs were recorded in *T. pratense* (Figure 5A) and *V. faba* (Figure 5B).

In both *T. pratense* and *V. faba*, the elR profiles changed in dependence on the distance from the stimulus (Figure 5C,D). In the target leaflet a strong and rapid initial depolarization (see convention in the Material and Methods Section 4.4) was followed by a long-lasting repolarization, which is characteristic of an EPW. Heat-induced EPWs were observed in 80% (*T. pratense*) to 100% (*V. faba*) of the target leaflets, in 25% (*T. pratense*) and 22% (*V. faba*) of the distant leaflets and no EPWs were detected in the systemic leaflets (Figure 5D). With increasing distance and increasingly complicated current distribution routes, the VP strength decreased in terms of voltage amplitude and duration until it was no longer detectable (Figure 5C,D). In *V. faba*, the EPW components AP and VP (AP + VP; Figure 5C,D) were more frequently distinguishable in distant leaflets (64%) than in target leaflets (0%; *p* < 0.001, Table S4). The same tendency was seen in *T. pratense* (distant leaflet 58%, target leaflet 10%; *p* = 0.057, Table S4). Pure APs were rarely detected (Figure 5D). In *V. faba*, the frequency of APs increased with the distance to the stimulus (0% in target leaflets, 14% in distant leaflets, 31% in systemic leaflets; *p* = 0.058, Table S4). The SPs could only be detected in *V. faba* in systemic leaflets (Figure 5D).

In both species, the time lapse between stimulus and start of the elR (i.e. depolarization) was significantly longer in distant leaflets (Figure 5E; both species *p* < 0.001, Table S5). The length of the depolarization decreased with the distance from the stimulus (*T. pratense p =* 0.029, *V. faba*; *p* < 0.001; Figure 5F; Table S5). In *T. pratense*, significantly shorter elR durations were observed in distant leaflets, whereas in *V. faba* such a reduction was only visible in systemic leaflets (Figure 5F). The elR propagation velocity decreased with the distance from the stimulus site as well (both plants *p* < 0.001; Figure 5G; Table S5).

## 3. Discussion

Several aspects of the biological relevance of forisomes have been determined over the past years. They prevent loss of phloem sap in case of damage, effectuate transient interruption of mass flow by reversible Ca^2+^-dependent dispersion in response to alarm signals (summarized in [13]) and are also involved in the defence against phloem feeders such as aphids [45]. To deepen the understanding of forisome functioning in planta, we investigated the relationships between SE and forisome dimensions, the forisome reactivity to electrical signalling and the type of elR at an interspecific level.

### 3.1. Forisome Dimensions are Related to the SE Diameters: Structure Relates to Function

There is a relationship between forisome length and width, the longer the forisomes the wider they were (Figure 1). However, at comparable length forisomes of *V. faba* and *M. sativa* are more slender than forisomes of *T. pratense* and *P. sativum* (Figure 1A). Strikingly this is not in accordance with the phylogenetic relations: *P. sativum* and *V. faba* belong to the tribe Fabeae and *M. sativa* and *T. pratense* to the tribe Trifolieae [28,46,47]. If forisome dimensions would comply with the phylogenetic relation, forisomes of species from one tribe should be more similar to each other. Since this is not the case, it can be assumed that factors other than phylogenetic relations are more important for determining forisome dimensions and a certain intraspecific bandwidth in forisome dimensions neither impairs forisome functioning nor hinders mass flow.

Interspecific variations in forisome size might be due to species-related amino acid sequences in SEO-F protein subunits [48,49]. Rose and colleagues showed for *M. truncatula* that ~45% of the full-length SEO-F1 sequence must remain conserved for forisome assembly [49]. This indicates an ability to modify several amino acids within a certain range without disturbing forisome assembly. By contrast, the correlation of forisome width to SE diameter was reported to be evolutionary conserved [30]. The species investigated here indeed showed a positive correlation between forisome *A_F_* and SE diameter. *M. sativa* and *T. pratense*, which are phylogenetically more closely related [47], shared an identical relationship of *A_F_* to the SE diameter (Figure 2 C1). These observations underline the functional importance of the relation between forisome size and SE size. Previous studies reported correlations between forisome dimensions and SE diameter [30,50]. Here, we show that the correlation also holds true for different species (Figure 1). It makes functional sense that forisome sizes enhance with increasing SE diameters towards the main veins. It is unknown how this adaptation is controlled and how size enlargement of forisomes is accomplished. We suspect the existence of a sensing system that tunes forisome size and SE diameter. Size enlargement may be achieved by an increased number of forisomettes (forisome building blocks; [51]) or by size enlargement of the building blocks themselves.

### 3.2. Forisome Dispersion is Linked to Forisome Dimensions

At comparable distances from the stimulus site, smaller forisomes (e.g., *A_F_* = 50 µm^2^) of *V. faba* and *T. pratense* dispersed faster (~25 s) than larger forisomes (e.g., *A_F_* = 150 µm^2^; ~40 s) of these species (Figure 3). Hence, the forisome size may impact on the dispersion mechanism. Forisome dispersion likely hinges on the presence of charged amino acid side groups [44,48]. The accumulation of counter-ions and subsequent water flux into the forisome structure, would lead to swelling of the forisome body [44]. Other models propose an electrostatic mechanism, in which Ca^2+^ ions disrupt forisome charge structures and repulsive forces increase the forisome volume [52,53,54]. Either hypothesis remains to be proven [48,49], but both imply Ca^2+^ influx into the forisome body. Large forisome bodies would likely need more Ca^2+^ ions than small ones to achieve a fully dispersed state. In addition, a larger forisome would offer a higher diffusion resistance and a longer time lapse until full dispersion. A lack of Ca^2+^ ions in the SE lumen or a higher internal resistance would explain partial dispersion of the large forisomes in *V. faba*.

### 3.3. Ca^2+^-Transmembrane Movements in Response to Stimuli May Differ between Legume Species

The duration of dispersion was neither correlated with the *A_F_* nor the distance from the stimulus site, but was species-specific. For example, forisomes of *T. pratense* stayed dispersed for a longer period than those of *V. faba* (ca. 300 s compared to ca. 200 s, Figure 3C). As the reactivity of forisomes depends on the Ca^2+^ availability within the SE, either the Ca^2+^-buffering capacity of the SE sap or the Ca^2+^ dissociation from the forisome could differ between the species. Assuming the Ca^2+^-dissociation constant calculated by Schwan and colleagues is similar for forisomes in different legume species [55], the amount of Ca^2+^ needed for full dispersion should be similar for equally sized forisomes of different species. The sustained dispersion of small *T. pratense* forisomes and to a certain extent *M. sativa* forisomes may therefore rely on buffering properties of the phloem sap and/or the activity of Ca^2+^ pumps [56,57], at the SE plasma membrane. A high pump density or activity would lead to a faster Ca^2+^ removal, shortening the period of forisome dispersion. This idea could be checked by investigating Ca^2+^-pump presence and pumping capacity in SEs of different legumes.

### 3.4. Increasing Distance from the Stimulus Site Impacts the Type of the Passing elR

**Figure 6:**
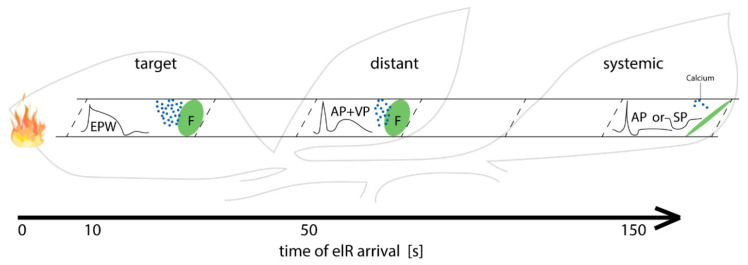
Schematic drawing of the events in target, distant and systemic leaflets after burning. In target leaflet the burning stimulus induces an electropotential wave (EPW = AP + VP) that results in the gating of Ca^2+^ -specific channels and therefore in forisome (F) dispersion. With increasing distance, different propagation velocities separate the recordings of AP and VP, while the VP-induced depolarization is reduced. In systemic leaflets the VP is completely lost and only the AP or SP can be detected, probably since too few Ca^2+^ ions are mobilized.

**Figure 7:**
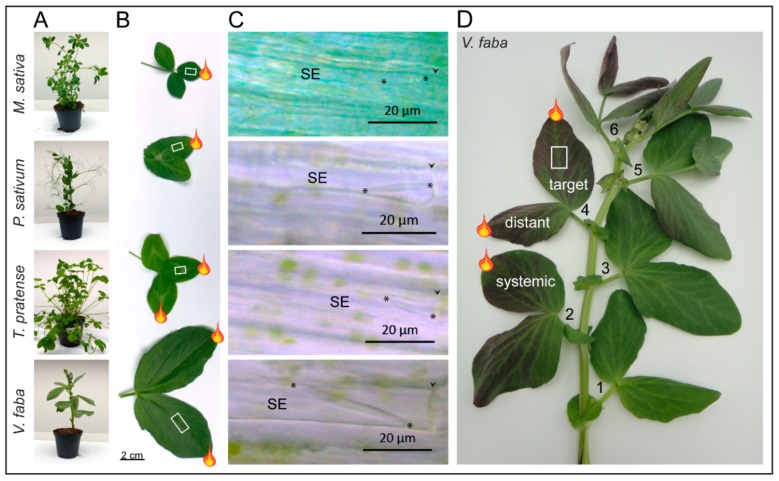
Overview of the plant species and experimental set-up. (**A**) Specimens of *M. sativa*, *P. sativum*, *T. pratense* and *V. faba* plants in use. The species possess compound leaves of various size and morphology (twin or triplet leaflets). (**B**) True to scale comparison of the respective leaves. The stimulated leaflets are labelled with a flame, the observation site is labelled with a white rectangle. (**C**) Micrographs of forisomes in the four legume species. Asterisks (*) indicate the forisome tips; arrow heads mark the location of a sieve plate. SE—Sieve element. (**D**) Detached *V. faba* plant stained with ink. One single incised stem section containing a major vascular bundle was submerged into an ink solution to illustrate the connectivity between leaves no. 2, 4 and 6. Mature leaves were numbered from oldest to youngest and leaf no. 4 was always used to cut the observation window (white rectangle). Flames indicate the different application sites of the heat stimulus.

The proportion of dispersing forisomes decreased with increasing distance from the stimulus site. This is in keeping with a retardation in Ca^2+^ signalling with increasing distance from the stimulus [58]. Because Ca^2+^ influx into SEs is triggered by an elR passage [13], we investigated the type of elRs in target, distant and systemic leaflets of the same orthostichy (Figure 6 and Figure 7D).

With increasing distance between target and systemic leaflets, the elR type ranged from an EPW (a merged AP + VP signal) in the target leaflet, separate AP + VP signals in distant leaflets to an AP, SP or no signal in systemic leaflets (Figure 5; also see schematic overview Figure 6). Previous studies also reported changing elRs along their way through the plant body [59,60]. The changing nature of elRs was ascribed to the resistance, offered by the numerous sieve plates that could attenuate or extinguish the signal [61] or to current leakage through plasmodesmata at the boundaries between SE and CC and in particular between CCs and parenchyma [13,62,63,64].

The type of passing elR had its bearing on the forisome reactivity (Figure 4 and Figure 6). The loss of VP in distant and systemic leaflets was correlated with a decreased chance of full forisome dispersion. This affirms the assumption that the change of elR type confers a different mode of information [59,60,61]. It also supports studies that favour VPs as the main trigger of forisome dispersion [37,65]. The heat-induced VP detected in the SE is initiated by a hydraulic pressure wave along the xylem [66,67,68] and depends on mechano-sensitive Ca^2+^ channels [13,69]. In consequence, these Ca^2+^ channels are responsible for forisome dispersion.

## 4. Material and Methods

### 4.1. Plant Cultivation

*Medicago sativa* L. cv. ‘Giulia’ (Appels Wilde Samen GmbH, Darmstadt, Germany), *Pisum sativum* L. cv. ‘Baccara’ (S.A.S. Florimond Desprez, Cappelle-en-Pévèle, France), *Trifolium pratense* L. cv. ‘Dajana’ (Appels Wilde Samen GmbH, Darmstadt, Germany), and *Vicia faba* L. cv. ‘The Sutton’ (Hazera Seeds UK Ltd., Lincolnshire, UK) plants were cultivated in 10 cm diameter plastic pots, with a standardized soil mixture of Klasmann Tonsubstrat and Klasmann Kultursubstrat TS1 (proportion 7:20; Klasmann–Deilmann GmbH, Geeste, Germany). The long-day conditions (16:8 h light:darkness) of the growth chamber (York Refrigeration, York, Pennsylvania, USA) were kept at an irradiance level of 100 to 150 µmol m^−2^ s^−1^ (Fluora lamps, Osram GmbH, Munich, Germany), a temperature regime between 20 to 22 °C and a relative humidity of 60 to 70%. Plants were used for experiments in their vegetative state before flowering (*M. sativa, T. pratense* 5–10 weeks and *P. sativum, V. faba* 4–9 weeks after germination).

### 4.2. General Experimental Set-Up

As a general approach, tips of leaflets at different positions and distances from the site of observation were burned with a focused match flame for 2 s, and forisome reactions were observed in the midvein of a defined leaflet. In *T. pratense* and *M. sativa* the central leaflet and in *V. faba* and *P. sativum* leaf number (no.) 4 (see Figure 7A) were used for studying local effects. For this burning and observation were done on the same leaflet (Figure 7B). Distant effects in *V. faba* and *T. pratense* were studied by burning a neighbouring leaflet of the observed one (Figure 7B). Systemic elR propagation and dispersion in *V. faba* were monitored after applying a heat stimulus to leaf no. 2 which is linked to leaf no. 4 via the vascular system, as documented by Eschrich [70] and depicted in Figure 7D. The relationship between forisome dimensions and the SE diameter or distance from the leaf tip, was analysed at various locations on the midvein of the leaf depending on the leaf size of each species using a ruler. *M. sativa* 1.0–2.0 cm; *P. sativum* 0.7–3.2 cm; *T. pratense* 0.5–3.8 cm; *V. faba* 1.1–6.5 cm. As forisomes at different locations (basal, central, apical) in the SE show different dispersion behaviours and times, only forisomes located proximally (basally) in SEs were considered for experiments [71], and each plant was used only once.

### 4.3. Preparation of Intact Plants for Microscopy

Microscopic examination of forisomes in intact SEs was largely identical to the established method described by Knoblauch and van Bel [17] and Furch et al. [71]. Cortical cell layers were removed with a razor blade from the lower site of the midvein down to the phloem so that intact SEs were exposed. Subsequently, the leaflet was attached to an objective slide with double-sided adhesive tape and the cut tissue was immersed in bathing medium (2 mM KCl, 1 mM CaCl_2_, 1 mM MgCl_2_, 50 mM mannitol, 2.5 mM MES/NaOH buffer, pH 5.7). The integrity of the phloem tissue was verified with a light microscope (AXIO Imager.M2, Zeiss, Jena, Germany) using a 40× water immersion objective (W-N Achroplan, Zeiss, Jena, Germany). Forisome responses to burning were investigated and recorded by a colour camera (AXIOCAM 503 color, Zeiss, Jena, Germany) one hour after phloem exposure to the bathing medium. Micrographs were processed with the ZEN software (Zeiss, Jena, Germany).

### 4.4. Preparation of Intact Plants for Electrophysiological Measurements

Voltage measurements were carried out on a vibration-stabilized bench enclosed by a Faraday cage. The electrodes consisted of a microelectrode holder (MEH1SF10 or MEH3S15; World Precision Instruments, Sarasota, FL, USA) and a glass capillary (tip diameter 1–2 µm; Hilgenberg GmbH, Malsfeld, Germany). The glass capillary tips were filled with 0.5 M KCl in 1% (w/v) agar to prevent an efflux of salt solution into the plant tissue and were backfilled with a 0.5 M KCl solution [60]. Two tips were blindly pierced (*T. pratense*: target leaflet at 2.5 cm and distant leaflet between 6–8 cm; *V. faba*: target leaflet at 3 and 6 cm, distant leaflet between 7–12 cm and systemic leaflet between 13–22 cm) into the midvein of a leaflet (see Figure 5A,B) and heat shocks applied after the resting potential had settled approximately 1–2 h after electrode settings. The microelectrodes were connected to a high input-impedance amplifier (KS-700; World Precision Instruments, Sarasota, FL, USA). The electrophysiological kinetics were recorded on an analogue pen chart recorder (W+W Recorder model 314, Darmstadt, Germany); the noise was reduced by a capacitor (1000 µF, 63 V). A reference electrode was inserted into the soil (in case of local measurements) or placed on a leaf tip inside a bathing solution [60].

**Convention:** According to classic intracellular measurements, a depolarization event is defined as a positive voltage change and a hyperpolarization event as a negative voltage change of a resting potential [42,60].

### 4.5. Calculation of the Approximate Forisome Surface Area in the Condensed State

Due to the spindle-shaped structure of forisomes, their simplified surface areas were estimated by assuming that a forisome is composed of two right circular cones. Thus the approximate surface area of a forisome (*A_F_*; Equation (2)) is two times the lateral surface area (*A_M_*; Equation (1)) of one cone. The height of the cone (*h*) corresponds to half the forisome length (*fl*) and the base radius (*r*) to half the forisome width (*fw*).

(1) $$A_{M}=r*\left(\sqrt{h^{2}+r^{2}}\right)*\pi$$

(2) $$A_{F}=\left(\frac{fw}{2}*\left(\sqrt{{\left(\frac{fl}{2}\right)}^{2}+{\left(\frac{fw}{2}\right)}^{2}}\right)*\;\pi \right)*2.$$

### 4.6. Illustration of the Vascular Connectivity

As described by Zimmermann et al. [60] one edge of the four-sided square stem of a *V. faba* plant was submersed in a commercial, coloured ink solution (Pelikan 4001 ink, brilliant red, Pelikan, Hanover, Germany) for at least 1 h. Translocation of ink via the xylem vessels throughout the plant visualized the preferential routes through the vascular bundles and revealed the orthostichy in 3D.

### 4.7. Statistics

Some observation windows made it possible to observe multiple forisomes at once. In those cases, the averages of the respective forisome and SE parameters were calculated and used for statistics as well as graphics. The dependencies of the forisome width from forisome length and plant species (Figure 1A); of the SE diameter from the position in the leaf and the plant species (Figure 1B); of the A_F_ from the SE diameter, the position in the leaf and the plant species (Figure 1C,D); and of the duration of the forisome dispersion from the distance to the burning site, A_F_ and the plant species (Figure 3C) were analysed with ANCOVAs. The plant species was set as categorical explanatory variable and forisome length, position of the forisome in the leaf, SE diameter and distance to the burning site and A_F_ as continuous explanatory variables. If necessary, the data was transformed to reach variance homogeneity and normality of the residuals. Differences between plant species were determined either with the TukeyHSD test (if the plant species was the only variable with a significant influence) or by factor level reduction [72]. The influence of the distance of the forisomes to the burning site, the forisome surface area and the plant species on the time until forisome dispersion (Figure 1A,B) was investigated using the generalized least squares method (gls from the nlme library [73]) with the varIdent variance structure to account for variance differences between plant species. The influence (*p*-values) of the explanatory variables was determined by sequential removal of explanatory variables from the full model, and comparison of the simpler with the more complex model with a likelihood ratio test [74]. Differences between factor levels were determined by factor level reduction [72].

Whether the ratio of fully dispersed forisomes to not or partially dispersed forisomes changed with the distance to the burning site (Figure 2) was analysed using a binomial generalized linear model (GLM). Corrected standard errors using quasi-GLM models were applied [74]. *p*-values were determined by removal of the explanatory variable from the full model, and comparison of the simpler with the more complex model with analysis of deviance tests [74].

Whether the ratio of fully dispersed forisomes to not or partially dispersed forisomes changed with the location of the burning site (target, distant or systemic leaflets; Figure 4) was analysed using the test for equality of proportions. The same test was applied to study whether the ratio of certain electrophysiological signals changed in different plant locations (target, distant or systemic leaflets, Figure 5). In case of significant differences, pairwise comparisons with a Bonferroni correction for multiple testing were performed. Whether the depolarization parameters (time until start, the duration and velocity of depolarization) changed with the location of the burning site was either analysed with Wilcoxon rank sum tests (in case of *T. pratense*) or with analyses of variance (in case of *V. faba*). In case of significant differences, the latter was followed by TukeyHSD test for pairwise comparisons.

### 4.8. Software

All statistical analyses were done using the R software, version 3.5.1 [75]. Figures were created using the Adobe Illustrator software (Adobe Systems Software Ireland Limited, Dublin, Ireland), version CS5.

## 5. Conclusions

In conclusion, our data support that forisome morphology vary within a certain range, but the relation of A_F_ to SE width is of functional importance and therefore, very likely evolutionarily conserved. A sufficient level of Ca^2+^ ions is required for a full forisome dispersion and the diffusion resistance correlates positively with the size of A_F_s that explains rising time lapses until dispersion. Only VPs above a critical threshold enable a Ca^2+^ influx which is capable of inducing complete forisome reactions (Figure 6). A possible approach for a future study to determine such critical threshold would be the application of different pressures or different concentrations of an osmotic solution like mannitol which induces VPs with different amplitudes [38].

## Data Availability

Not applicable.

## Supplementary Materials

Supplementary materials can be found at https://www.mdpi.com/1422-0067/22/2/492/s1. Table S1. Statistical values for the analysis of morphological traits of forisomes according to several explanatory variables. Table S2. Statistical values for the analysis of the ratio between fully dispersed and not fully dispersed forisomes in the target leaflet according to the distance to the stimulation site. Table S3. Statistical values for the analysis of the time elapsed from burning to the full dispersion of forisomes and the duration of dispersion according to the traveling distance, forisome surface area and plant species. Table S4. Statistical values for the analyses of proportions of electrophysiological reaction in different leaflets of *T. pratense* and *V. faba*. Table S5. Statistical values for the analyses of dependence of the depolarization parameters on the site of the stimulus.

## Abbreviations

A_F_	forisome surface area
A_M_	lateral surface area
AP	action potential
CC	companion cell
cv.	cultivar
elR	electrophysiological reaction
EPW	electropotential wave
fl	forisome lenght
fw	forisome width
h	Height of the cone
no.	number
r	base radius
SE	sieve element
SEO	sieve element occlusion
SEOR	sieve element occlusion related
SEO-F	sieve element occlusion by forisome
SP	system potential
VP	variation potential
