# Supplementary material for: Species-Specific and Distance-Dependent Dispersive Behaviour of Forisomes in Different Legume Species
Source: Int J Mol Sci. 2021 Jan 6;22(2):492. doi: 10.3390/ijms22020492 (PMC7825422; doi:10.3390/ijms22020492)
Supplement: Supplementary file 1 [file ijms-22-00492-s001.pdf]

Supplemental Material

Table S1: Statistical values for the analysis of morphological traits of forisomes according to several explanatory variables (corresponding to Figure 1).

Trait / Transformation	Explanatory variables	*F*-value	*P*-value
**Forisome width**			
	Forisome length : Plant	0.726	0.537
	Forisome length	896.620	**< 0.001**
	Plant	16.890	**< 0.001**
**SE diameter (sqrt transformed)**			
	Window distance : Plant	1.610	0.187
	Window distance	783.500	**< 0.001**
	Plant	222.800	**< 0.001**
**Forisome surface area (sqrt transformed)**			
	SE diameter : Window distance : Plant	0.108	0.955
	SE diameter : Plant	1.034	0.378
	Window distance : Plant	1.452	0.228
	SE diameter : Window distance	6.597	**0.011**
	SE diameter	1459.418	**< 0.001**
	Window distance	18.178	**< 0.001**
	Plant	34.798	**< 0.001**

Significant *P*-values are given in bold. sqrt – square root; SE – sieve element; window distance – the distance from the leaf tip to the observation window in the leaf.

Table S2: Statistical values for the analysis of the ratio between fully dispersed and not fully dispersed forisomes in the target leaflet according to the distance to the stimulation site (corresponding to Figure 2).

Plant	*F*-value	*P*-value
M. sativa	3.470	0.077
P. sativum	0.535	0.472
T. pratense	2.089	0.154
V. faba	16.695	< **0.001**

The degrees of freedom were 1 in all cases. Significant *P*-values are given in bold.

Table S3: Statistical values for the analysis of the time elapsed from burning to the full dispersion of forisomes and the duration of dispersion according to the traveling distance, forisome surface area (A_F) and plant species (corresponding to Figure 3).

Trait	Explanatory variables	L-ratio / *F-value*	*P*-value
**Elapsed time until dispersion***			
	Traveling distance* : A_F*: Plant	7.212	0.066
	Traveling distance* : A_F*	0.259	0.611
	Traveling distance* : Plant	5.189	0.158
	A_F*: Plant	15.263	**0.002**
	Plant	3.718	0.294
	A_F*	1.096	0.295
	Traveling distance*	46.949	**< 0.001**
**Duration of dispersion***			
	Traveling distance : A_F: Plant	*1.360*	0.258
	Traveling distance : A_F	*0.060*	0.807
	Traveling distance : Plant	*1.507*	0.216
	A_F: Plant	*0.570*	0.635
	Plant	*3.966*	**0.009**
	A_F	*0.913*	0.341
	Traveling distance	*1.275*	0.261

Significant *P*-values are given in bold. For the analysis of the elapsed time until dispersion the varIdent variance structure was used to account for the variance heterogeneity of the residuals introduced by the plant species. Depending which statistical test was used Likelihood (L)-ratios or *F*-values are given. *F*-values are given in italics. * indicates the ln-transformation of the respective parameter. Traveling distance – distance from the burning site to the observation window.

Table S4: Statistical values for the analyses of proportions of electrophysiological reaction in different leaflets of *T. pratense* and *V. faba* (corresponding to Figure 5 D).

Electrophysiological reaction	*Trifolium pratense*		*Vicia faba*	
	Chi^2	*P*-value	Chi^2	*P*-value
**EPW**	4.583	**0.032**	41.539	**< 0.001**
**AP + VP**	3.616	0.057	17.455	**< 0.001**
**AP**	n.a.	n.a.	7.494	0.058

Only electrophysiological reactions which occurred more than 5 times were statistically analysed. Significant *P*-values are given in bold. Degrees of freedom for *T. pratense* = 1, for *V. faba* = 3. n.a. – not applicable.

Table S5: Statistical values for the analyses of dependence of the depolarization parameters on the site of the stimulus (corresponding to Figure 5 E).

**Depolarization parameter**	**Plant**	**Transformation**	***W* / F-value**	***P-value***
**Start of depolarization**	*T. pratense*	n.a.	*110*	**< 0.001**
	V. faba	ln	84.350	**< 0.001**
**Duration**	*T. pratense*	n.a.	*24*	**0.029**
	V. faba	ln	6.801	**< 0.001**
**Velocity**	*T. pratense*	n.a.	*~ 0*	**< 0.001**
	V. faba	ln	25.420	**< 0.001**

Significant *P*-values are given in bold. Depending which statistical test was used *W*- or F-values are given. *W-values* are given in italics. n.a. – not applicable.
